# Sensor Fusion Based on an Integrated Neural Network and Probability Density Function (PDF) Dual Kalman Filter for On-Line Estimation of Vehicle Parameters and States

**DOI:** 10.3390/s17050987

**Published:** 2017-04-29

**Authors:** Leandro Vargas-Melendez, Beatriz L. Boada, Maria Jesus L. Boada, Antonio Gauchia, Vicente Diaz

**Affiliations:** 1 Mechanical Engineering Department, Research Institute of Vehicle Safety, Universidad Carlos III de Madrid, Avda. de la Universidad 30, 28911 Madrid, Spain; lvargas@ing.uc3m.es (L.V.-M.); mjboada@ing.uc3m.es (M.J.L.B.); vdiaz@ing.uc3m.es (V.D.); 2 Mechanical Engineering-Engineering Mechanics Department, Michigan Tech University, 1400 Townsend Drive, Houghton, MI 49931, USA; antonio@mtu.edu

**Keywords:** vehicle dynamics, dual Kalman filter, probability density function (PDF) truncation, state estimation, parameter estimation, vehicle roll angle, sensor fusion

## Abstract

Vehicles with a high center of gravity (COG), such as light trucks and heavy vehicles, are prone to rollover. This kind of accident causes nearly 33% of all deaths from passenger vehicle crashes. Nowadays, these vehicles are incorporating roll stability control (RSC) systems to improve their safety. Most of the RSC systems require the vehicle roll angle as a known input variable to predict the lateral load transfer. The vehicle roll angle can be directly measured by a dual antenna global positioning system (GPS), but it is expensive. For this reason, it is important to estimate the vehicle roll angle from sensors installed onboard in current vehicles. On the other hand, the knowledge of the vehicle’s parameters values is essential to obtain an accurate vehicle response. Some of vehicle parameters cannot be easily obtained and they can vary over time. In this paper, an algorithm for the simultaneous on-line estimation of vehicle’s roll angle and parameters is proposed. This algorithm uses a probability density function (PDF)-based truncation method in combination with a dual Kalman filter (DKF), to guarantee that both vehicle’s states and parameters are within bounds that have a physical meaning, using the information obtained from sensors mounted on vehicles. Experimental results show the effectiveness of the proposed algorithm.

## 1. Introduction

One of the main causes of accidents in road transport is the loss of vehicle stability. Particularly, vehicles with a high center of gravity (COG), such as light trucks and heavy vehicles, are prone to rollover. Rollover accidents cause nearly 33% of all deaths from passenger vehicle crashes. Nowadays, these kinds of vehicles are incorporating roll stability control (RSC) systems to improve their lateral stability and handling. Most of the RSC systems require the vehicle roll angle as a known input variable to predict the lateral load transfer [1,2]. The vehicle roll angle can be directly measured by a dual antenna global positioning system (GPS), but it is expensive. For this reason, many researchers have focused on vehicle roll angle estimation [3,4,5,6,7]. One of the main techniques employed to estimate roll angle is through sensor fusion. In [6], the vehicle roll angle is estimated integrating the information from a lateral accelerometer and suspension deflection sensors. Since one of the main disadvantages of using suspension deflection sensors is cost, they are not currently installed in vehicles. Besides, results show that the estimation of vehicle roll angle from this technique is not very accurate compared to other methods [7].

In [8,9], GPS and onboard vehicle sensors are employed to measure the vehicle roll angle. The drawback of using a GPS device is the difficulty in achieving accurate readings since visibility of satellites in both urban and forest driving environments [10] can hamper GPS performance.

The Kalman filter is a well known established method used to fuse the information obtained from different sensors. In [7,10,11], the Kalman filter estimates the vehicle roll angle. However, these algorithms do not consider that the parameters of the vehicle model can change, since they might be time-dependent. It is important to highlight that knowledge of the vehicle’s parameters values is essential to obtain an accurate vehicle response. Whereas some parameters, such as vehicle mass and wheelbase can be easily obtained, other parameters, such as roll stiffness and roll damping coefficient, have to be estimated through an identification process. Besides, some of these parameters can vary over time, hence a model adjustment through time variation of parameters along with state variables is crucial.

In some works, the dual Kalman filter (DKF) is used to simultaneously obtain an estimation of states and of parameters [12,13,14,15]. The disadvantage of these works is that neither states nor parameters are constrained. The solution to this problem is very complex and sometimes non-physical meaning solutions can be obtained. Since the problem to be solved has a large set of states and parameters, the available sensor measurements are small compared to the amount of existing states/parameters and the vehicle model is a non-linear model. Hence, it is necessary to consider constraints for both states and parameters. Some methods have previously been proposed to deal with constraints in the Kalman filter, such as the projection method [16,17,18] and the probability density function (PDF) truncation method [19,20,21,22]. In [22], a comparison between the projection method and the PDF trunction method is performed for the estimation of the road bank angle and vehicle’s parameters. Results show that the PDF trunction method has a better performance than the projection method. This paper uses a dual antenna GPS in order to measure the total vehicle roll angle and, as previously mentioned, this is a costly method.

The novelty of this paper is to design an observer to estimate on-line the vehicle roll angle and vehicle’s parameters. This observer integrates neural networks (NN) and a PDF dual Kalman filter. NN provides to the PDF dual Kalman filter a “pseudo-roll angle” which is used as a measurement in the Kalman filter. The design of this observer:

1. estimates, simultaneously and on-line, the vehicle’s states and parameters.
2. uses a simplified vehicle model,
3. is useful in all kinds of environments (tunnels, urban and forested driving environments),
4. uses signals of sensors installed on-board in current vehicles,
5. takes into consideration both the measurements and model errors.

This paper is organized as follows. In Section 2, the vehicle model used in discrete time-space model is described. The advantage of this model is that it is a simplified vehicle model. In Section 3 the proposed estimator for vehicle parameters and states is described. This estimator uses NN to calculate the “pseudo-roll angle” which is introduced as an input into the constrained DKF. The DKF simultaneously estimates the parameters and the states. The PDF truncation algorithm is used in order to limit the vehicle parameter values to their physical limits. Experimental results are shown in Section 4 and a discussion of them. Finally, the summary and conclusions are given in Section 5.

## 2. Vehicle Model

**Figure 1:**
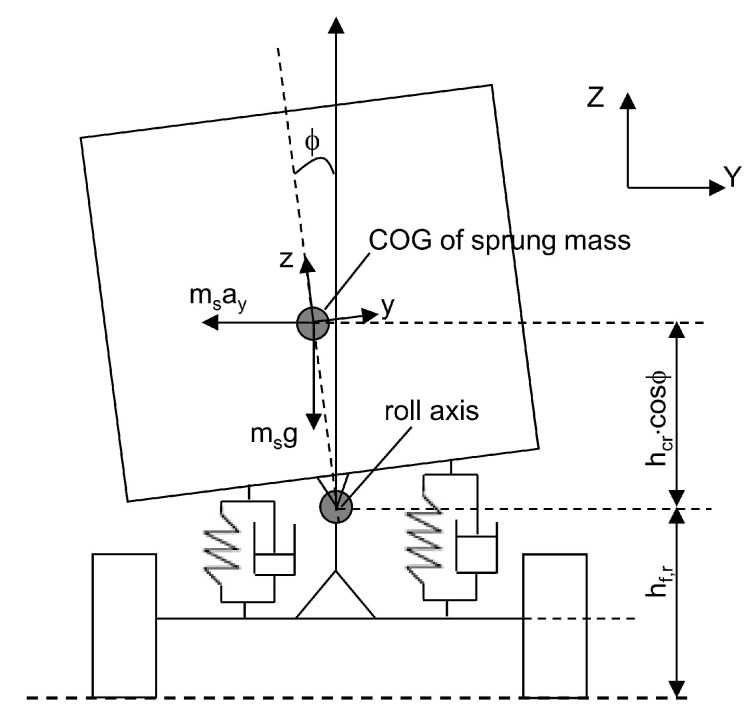
1-DOF vehicle model. COG: center of gravity.

A 1-DOF vehicle model is used in the Kalman filter. This model is widely adopted to describe the vehicle roll motion (Figure 1). In the model, a fixed coordinate system (x, y, z) is employed in order to describe the vehicle roll motion. It is assumed that the vehicle sprung mass rotates around the roll center of the vehicle. The vehicle’s roll dynamic motion is governed by the following differential equation [7]:

(1) $$I_{xx}\ddot{\phi }+C_{R}\dot{\phi }+K_{R}\phi =m_{s}a_{y}h_{cr}+m_{s}h_{cr}g\sin\left(\phi \right)$$

where ϕ is the vehicle roll angle, $I_{xx}$ is the sprung mass moment of inertia with respect to the roll axis, $m_{s}$ is the sprung mass, $h_{cr}$ is the sprung mass height about the roll axis, $C_{R}$ represents the total torsional damping, $K_{R}$ is the stiffness coefficient, $a_{y}$ represents the lateral acceleration at the vehicle center of gravity (COG) and *g* is the acceleration due to gravity.

If the roll angle is assumed to be small, the equation that relates the vehicle lateral acceleration, $a_{y}$, and the lateral acceleration measured by the accelerometer, $a_{ym}$ is:

(2) $$a_{y}\approx a_{ym}-g\phi$$

Additionally, if the pitching and the bounding motions of the sprung mass are assumed to be neglected and the road bank angle is considered to be small, then, the vehicle roll rate, ϕ, is equal to the roll rate given by the rate sensor, $\phi_{m}$:

(3) $$\dot{\phi }\approx {\dot{\phi }}_{m}$$

In this work, a non-descriptive model is assumed, ${\ddot{a}}_{y}=0$ [23,24]. Then, the vehicle model is represented in the time domain by means of a discrete time state-space model:

(4) $$\begin{array}{c} x_{s,k}=A_{d}x_{s,k-1}+w_{k}\\ y_{k}=H_{s}x_{s,k}+v_{k} \end{array}$$

where $x_{s,k}=[a_{y},{\dot{a}}_{y},\phi ,\dot{\phi }]$ represents the state vector, $A_{d}$ is the state evolution matrix:

$$A_{d}=\left[\begin{array}{cccc} 1 & T_{s} & 0 & 0\\ 0 & 1 & 0 & 0\\ 0 & 0 & 1 & T_{s}\\ T_{s}m_{s}\frac{h_{cr}}{I_{xx}} & 0 & T_{s}\frac{m_{s}gh_{cr}-K_{R}}{I_{xx}} & 1-T_{s}\frac{C_{R}}{I_{xx}} \end{array}\right]$$

$T_{s}$ is the sample time, and $H_{s}$ is the observation matrix:

$$H_{s}=\left[\begin{array}{cccc} 1 & 0 & g & 0\\ 0 & 0 & 1 & 0\\ 0 & 0 & 0 & 1 \end{array}\right]$$

$y={\left[a_{ym},\phi ,\dot{\phi }\right]}^{T}$ is the measurement vector, $w_{k}$ and $v_{k}$ are the state disturbance and the observation noise vectors, respectively, that are assumed to be Gaussian, uncorrelated and zero mean:

(5) $$\begin{array}{c} w_{k}∼N\left(0,Q\right)\\ v_{k}∼N\left(0,R\right) \end{array}$$

where Q is the covariance matrix of the process noise and R is the covariance matrix of the measurement noise.

## 3. Vehicle’s Parameters and Roll Angle Estimation

**Figure 2:**
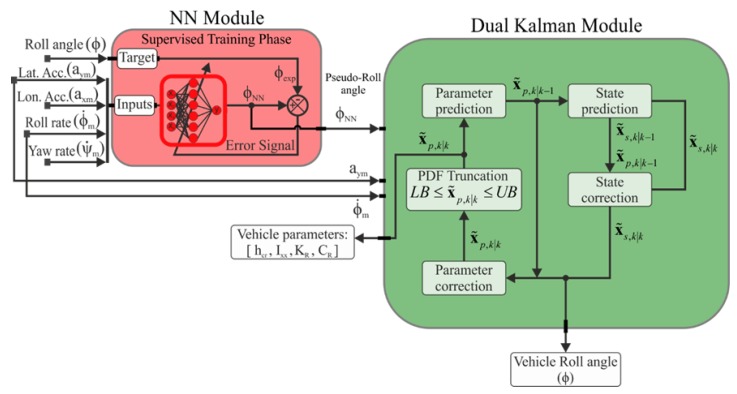
Estimator architecture. NN: neural network; PDF: probability density function.

The architecture of the proposed estimator is given in Figure 2. The estimator is based on a neural network (NN) combined with a PDF truncation DKF in order to on-line estimate the vehicle roll angle and the vehicle’s parameters. The vehicle’s parameters to be estimated are the moment of inertia of the sprung mass with respect to the roll axis, $I_{xx}$, the total torsional damping and stiffness coefficients of the roll motion of the vehicle, $K_{R}$ and $C_{R}$, respectively, and the height of the sprung mass about the roll axis, $h_{cr}$. Both vehicle’s parameters and roll angle are estimated through the fusion of information provided by different sensors, such as the longitudinal and lateral accelerations, $a_{xm}$ and $a_{ym}$, the roll rate ${\dot{\phi }}_{m}$ and the yaw rate ${\dot{\psi }}_{m}$.

The observer architecture is formed by two blocks: the NN block and the PDF DKF block. The NN block estimates a “pseudo-roll angle” from signals which are easily measured by an inertial measurement unit (IMU).

Note that the cost of IMU has decreased in recent years. A detailed description about the training of the NN and results obtained can be found in our previous work [7]. One of the advantage of using the NN module is that the “pseudo-roll angle” is directly estimated from IMU measurements, so that no integration is carried out to get this data. This “pseudo-roll angle” is fed to the new proposed DKF module as an input. The proposed method achieves good roll angle estimations by taking into consideration the vehicle non-linearities and parameter variations for every time step. The proposed method differs from [7], since a separate state-space system definition is employed to estimate the states and to predict the vehicle parameters, some of them being time-dependent. This feature greatly increases the time-domain state estimator.

### 3.1. DKF Module

The purpose of the PDF DKF module is to estimate the vehicle’s parameters and the states of a linear vehicle model defined in Equation (4) by means of two Kalman filters. The Kalman filter is a mathematical tool that is used for stochastic estimation from data that include a substantial amount of noise and unobserved states in the system which must be estimated. Moreover, the Kalman filter allows reducing accumulated errors using sensor measurements.

In this work, we consider a separate state-space formulation for states and parameters [25]. The main advantage of using a separate state-space formulation is that it is possible to switch off the parameter estimator, once a sufficiently good set of estimates for the parameters have been found [26]. This increases the performance of the state estimator, since it reduces the parameter uncertainties as well as disturbances arising from the varying model parameters.

The DKF algorithm has the following recursive procedure:

1. Parameter prediction:
  (6) $${\tilde{x}}_{p,k|k-1}={\tilde{x}}_{p,k-1|k-1}$$
  (7) $$P_{p,k|k-1}=P_{p,k-1|k-1}+Q_{p}$$
2. State prediction:
  (8) $${\tilde{x}}_{s,k|k-1}=A_{d}\left({\tilde{x}}_{p,k|k-1}\right){\tilde{x}}_{s,k-1|k-1}$$
  (9) $$P_{s,k|k-1}=A_{d}\left({\tilde{x}}_{p,k|k-1}\right)P_{s,k-1|k-1}A_{d}{\left({\tilde{x}}_{p,k|k-1}\right)}^{T}+Q_{s}$$
3. State correction:
  (10) $$K_{s,k}=P_{s,k|k-1}H_{s}^{T}{\left[H_{s}P_{s,k|k-1}H_{s}^{T}+R_{s}\right]}^{-1}$$
  (11) $${\tilde{x}}_{s,k|k}={\tilde{x}}_{s,k|k-1}+K_{s,k}\left[y_{k}-H_{s}{\tilde{x}}_{s,k|k-1}\right]$$
  (12) $$P_{s,k|k}=\left[I-K_{s,k}H_{s}\right]P_{s,k|k-1}$$
4. Parameter correction:
  (13) $$K_{p,k}=P_{p,k|k-1}J^{T}{\left[{\mathrm{JP}}_{p,k|k-1}J^{T}+R_{p}\right]}^{-1}$$
  (14) $${\tilde{x}}_{p,k|k}={\tilde{x}}_{p,k|k-1}+K_{p,k}\left[y_{k}-H_{s}{\tilde{x}}_{s,k|k-1}\right]$$
  (15) $$P_{p,k|k}=\left[I-K_{p,k}J\right]P_{p,k|k-1}$$

where ${\tilde{x}}_{p,k}=\left[h_{cr},I_{xx},K_{R},C_{R}\right]$ is the parameter vector, ${\tilde{x}}_{s,k}=\left[a_{y},{\dot{a}}_{y},\phi ,\dot{\phi }\right]$ is the state vector, $P_{s}$ and $P_{p}$ are the error covariances matrices for states and parameters, respectively. $K_{s}$ and $K_{p}$ are the Kalman gain matrices for states and parameters, respectively. J is the Jacobian matrix of parameter estimator given by:

(16) $$\begin{array}{c} J=\left[\begin{array}{cccc} \frac{\partial a_{ym}}{\partial h_{cr}} & \frac{\partial a_{ym}}{\partial I_{xx}} & \frac{\partial a_{ym}}{\partial K_{R}} & \frac{\partial a_{ym}}{\partial C_{R}}\\ \;\frac{\partial \phi }{\partial h_{cr}} & \frac{\partial \phi }{\partial I_{xx}} & \frac{\partial \phi }{\partial K_{R}} & \frac{\partial \phi }{\partial C_{R}}\\ \;\frac{\partial \dot{\phi }}{\partial h_{cr}} & \frac{\partial \dot{\phi }}{\partial I_{xx}} & \frac{\partial \dot{\phi }}{\partial K_{R}} & \frac{\partial \dot{\phi }}{\partial C_{R}} \end{array}\right]\\ =\left[\begin{array}{cccc} 0 & 0 & 0 & 0\\ 0 & 0 & 0 & 0\\ \left(\frac{T_{s}m_{s}}{I_{xx}}a_{y}+\frac{T_{s}m_{s}g}{I_{xx}}\phi \right) & \left(-\frac{T_{s}m_{s}h_{cr}}{I_{xx}^{2}}a_{y}-\frac{T_{s}\left(m_{s}gh_{cr}-K_{R}\right)}{I_{xx}^{2}}\phi +\frac{T_{s}C_{R}}{I_{xx}^{2}}\dot{\phi }\right) & \left(-\frac{T_{s}}{I_{xx}}\phi \right) & \left(-\frac{T_{s}}{I_{xx}}\dot{\phi }\right) \end{array}\right] \end{array}$$

Since the states and parameters estimators depend on the same output vector, $y_{k}$, the covariance matrices of the measurement noise are the same:

(17) $$R_{s}=R_{p}=\left[\begin{array}{ccc} \sigma_{a_{ym}}^{2} & 0 & 0\\ 0 & \sigma_{\phi_{NN}}^{2} & 0\\ 0 & 0 & \sigma_{\dot{\phi }}^{2} \end{array}\right]$$

where $\sigma_{a_{ym}}=0.01$ m/s${}^{2}$, $\sigma_{\phi_{NN}}=0.01$${}^{\circ }$ and $\sigma_{{\dot{\phi }}_{m}}=0.01$${}^{\circ }$/s are the noise covariances associated with the measurement sensors.

$Q_{s}$ is the process noise covariance matrix of the state estimator:

(18) $$Q_{s}=R_{0}I$$

Good results are obtained when $R_{0}$ takes a large value [22,26]. In this work, $R_{0}=100,000,000$. Finally, $Q_{p}$ is the process noise covariance matrix of the parameter estimator:

(19) $$Q_{p}=\left[\begin{array}{cccc} \sigma_{h_{cr}}^{2} & 0 & 0 & 0\\ 0 & \sigma_{I_{xx}}^{2} & 0 & 0\\ 0 & 0 & \sigma_{K_{R}}^{2} & 0\\ 0 & 0 & 0 & \sigma_{C_{R}}^{2} \end{array}\right]$$

$\sigma_{h_{cr}}$, $\sigma_{I_{xx}}$, $\sigma_{K_{R}}$ and $\sigma_{C_{R}}$ are taken as a 1% of the initial values of $h_{cr}$, $I_{xx}$, $K_{R}$ and $C_{R}$, respectively [22,26].

### 3.2. PDF Truncation Approach

Even though the proposed dual state space definition for states and variables increase the overall performance, there is also a high number of estimated variables that pose an additional difficulty, thus resulting in a complex problem to solve. For this reason, sometimes, the obtained values might be outside the physical limit boundaries. To avoid this situation, the DKF has to impose constraints in states/parameters. In order to reduce the computational cost, in this work, only the parameter constraints have been considered. These constraints are taken into account using a PDF truncation approach [16,18,20] which is incorporated to the DKF defined in Section 3.1.

The objective is to estimate ${\tilde{x}}_{p,k}$ which is defined as a Gaussian random vector with mean $x_{p,k}$ and covariance $P_{p,k|k}$. At time *k*, for each parameter *p*, the constraints are expressed as:

(20) $$a_{i}\leq D_{i}^{T}{\tilde{x}}_{p,k\left|k\right.}\leq b_{i}\;\;\;\;\;\;\;\;\;\;i=1,\ldots ,p$$

where $a_{i}$ and $b_{i}$ represent the lower and upper bound for each vehicle’s parameter, respectively. $D_{i}$ is an *p*-element column vector comprised entirely of zeros, except that its *i* element is 1.

The PDF truncation algorithm is summarized as follows:

1. Initialization, *i* = 0
  (21) $$\begin{array}{c} {\tilde{x}}_{p,i}\left(k\right)={\tilde{x}}_{p,k|k}\\ P_{p,i}\left(k\right)=P_{p,k|k} \end{array}$$
  ${\tilde{x}}_{p,k|k}$ and $P_{p,k|k}$ are obtained from the DKF module (see Section 3.1).
2. For *i* = 1 to *i* = *p*, the mean and variance of the parameter state are calculated by means of the following equations:
  (22) x˜p,i+1(k)=T·W12·SiT·z˜i,k+x˜p,i(k)
  (23) Pp,i+1(k)=T·W12·SiT·cov(z˜i,k)·Si·W12·TT
  $T_{i}$ and $W_{i}$ are calculated from the Jordan canonical decomposition of $P_{p,i}\left(k\right)$ (see Appendix A), that fulfills the condition stated by the equation:
  (24) $$T_{i}\cdot W_{i}\cdot T_{i}^{T}=P_{p,i}\left(k\right)$$
  $S_{i}$ is an orthogonal matrix obtained by using Gram–Schmidt orthogonalization that satisfies (see Appendix B):
  (25) $$S_{i}W_{i}^{1/2}T_{i}^{T}D_{i}\left(k\right)={\left[\begin{array}{cccc} {\left(D_{i}^{T}\left(k\right)P_{i,k}\left(k\right)D_{i}\left(k\right)\right)}^{1/2} & 0 & ... & 0 \end{array}\right]}^{T}$$
  The vector $z_{i,k}$ has mean 0 and an identity covariance matrix. Hence, its elements are statistically independent of one another. Only the first element of $z_{i,k}$ is constrained, therefore, the PDF truncation is reduced to a one-dimensional truncation:
  (26) $$z_{i,k}={\left[\begin{array}{cccc} \mu_{i} & 0 & ... & 0 \end{array}\right]}^{T}$$
  (27) $$\mathrm{cov}\left(z_{i,k}\right)=diag\left(\sigma_{i}^{2},1,...,1\right)$$
  where $\mu_{i}$ is the truncated mean value given by the following expression:
  (28) $$\mu_{i}=\alpha_{i}\left[\exp\left(-c_{{}_{i,k}}^{2}/2\right)-\exp\left(-d_{{}_{i,k}}^{2}/2\right)\right]$$
  and $\sigma_{i}^{2}$ is the truncated covariance given by the following equation:
  (29) $$\sigma_{{}_{i}}^{2}=\alpha_{i}\left[\exp\left(-c_{{}_{i,k}}^{2}/2\right)\left(c_{i,k}-2\mu_{i}\right)-\exp\left(-d_{{}_{i,k}}^{2}/2\right)\left(d_{i,k}-2\mu_{i}\right)\right]+\mu_{{}_{i}}^{2}+1$$
  where
  (30) $$\alpha_{i}=\frac{\sqrt{2}}{\sqrt{\pi }\left[erf\left(\frac{d_{i,k}}{\sqrt{2}}\right)-erf\left(\frac{c_{i,k}}{\sqrt{2}}\right)\right]}$$
  The truncated PDF is normalized to achieve a unity area, and the fist element of $z_{i,k}$ is constrained:
  (31) $$c_{i,k}\leq \left[\begin{array}{cccc} 1 & 0 & ... & 0 \end{array}\right]z_{i,k}\leq d_{i,k}$$
  so that,
  (32) $$c_{i,k}=\frac{a_{i}-\left(D_{i}^{T}\left(k\right){\tilde{x}}_{p,i}\left(k\right)\right)}{{\left(D_{i}^{T}\left(k\right)P_{p,i}\left(k\right)D_{i}\left(k\right)\right)}^{1/2}}$$
  (33) $$d_{i,k}=\frac{b_{i}-\left(D_{i}^{T}\left(k\right){\tilde{x}}_{p,i}\left(k\right)\right)}{{\left(D_{i}^{T}\left(k\right)P_{p,i}\left(k\right)D_{i}\left(k\right)\right)}^{1/2}}$$
3. Finally, the final constrained parameter estimate and covariance at time *k* is given by:
  (34) $${\tilde{x}}_{p,k\left|k\right.}={\tilde{x}}_{p,p}\left(k\right)$$
  (35) $$P_{p,k\left|k\right.}=P_{p,p}\left(k\right)$$

## 4. Experimental Results and Discussion

**Figure 3:**
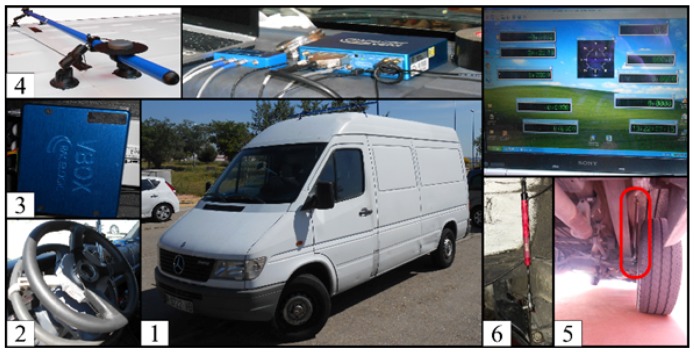
Test vehicle equipped with different sensors.

A Mercedes Sprinter is used for this research, as depicted in Figure 3. For the experimental results, different sensors were installed in the vehicle, such as an MSW 250 Nm steering angle sensor from Kistler (2), a Vbox 3i dual antenna from Racelogic (3) which utilizes two GPS/GLONASS antennas (4) and an inertial measurement unit (IMU). The IMU was installed close to the vehicle COG. The two antennas were installed on the Mercedes’s roof, placed at an angle of 90 degrees relative to the vehicle true heading, allowing the system to measure the roll angle. This roll angle value has been considered as ground truth and has been used to validate the proposed estimator.

To prove the performance of the proposed algorithm, a comparison between the estimation of “pseudo-roll angle” given by [6] and given in this work is carried out. For this reason, four linear potentiometers, (5) and (6) in Figure 3 (Type SA-LP075 from 2D-Data to record data from the front suspension), as well as two sensors (Type LVDT MTN from Monitran for the rear suspension), were additionally mounted on the vehicle. In [6], the measurements obtained from these sensors were used to estimated the vehicle roll angle:

(36) $$\phi_{DEF}=\frac{\left(\Delta_{11}-\Delta_{12}+\Delta_{21}-\Delta_{22}\right)}{2e}-\frac{m_{v}a_{ym}h}{k_{t}}$$

where $k_{t}$ is the roll tire stiffness whose value is 607,500 Nm/rad, *h* is the height of vehicle COG whose value is 0.98 m, *e* is the vehicle track whose value is 1.634 m, $m_{v}$ is the vehicle mass whose value is 2150 kg, $\Delta_{ij}$ is the suspension deflection and $a_{ym}$ is lateral acceleration given by the sensor.

For the vehicle used in experiments, the valid parameter values lie between:

$${\left[0.1,500,10^{4},10^{4}\right]}^{T}\leq {\left[h_{c}r,I_{xx},K_{R},C_{R}\right]}^{T}\leq {\left[0.4,1000,10^{5},10^{5}\right]}^{T}$$

In the following subsections, different experimental cases are conducted in order to show the performance of the algorithm proposed.

### 4.1. Case 1: Combination of Slalom and J-Turn Maneouvres

**Figure 4:**
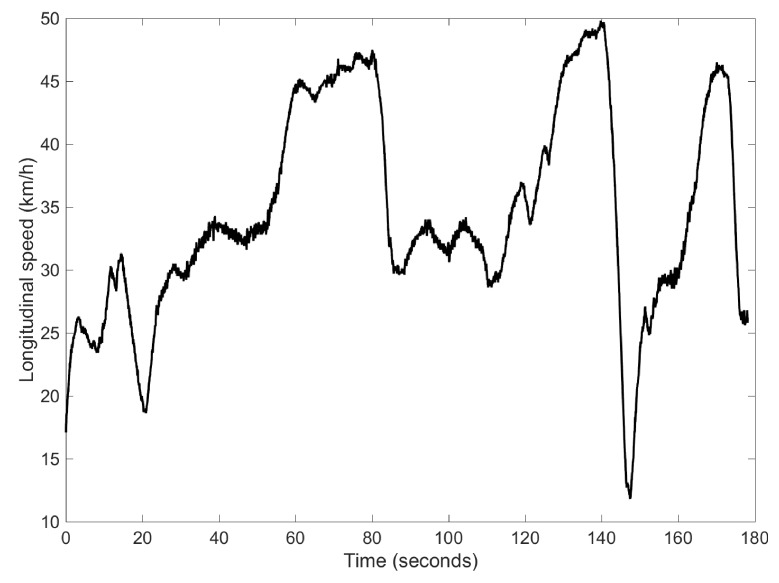
Speed profile for Case 1.

**Figure 5:**
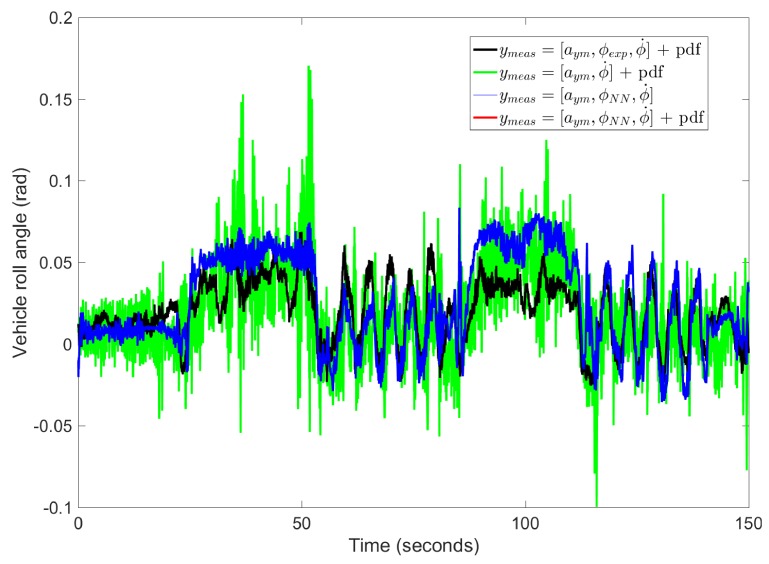
Vehicle roll angle for Case 1: Experimental data with dual antenna (black points), estimated vehicle roll angle without considering the “pseudo-roll angle” (red points) and estimated vehicle roll angle considering the “pseudo-roll angle” from NN (blue points).

The first experiment carried out is a combination of Slalom and J-turn maneouvres on a dry pavement. The vehicle speed profile is shown in Figure 4. In order to prove the need to use the information of the “pseudo-roll angle” provided by the NN, a comparison between the results obtained from the proposed algorithm using or not the “pseudo-roll angle” as an input in the DKF is shown in Figure 5, $y_{meas}=\left[a_{ym},\phi_{NN},\dot{\phi }\right]$ and $y_{meas}=\left[a_{ym},\dot{\phi }\right]$, respectively. In this figure, the vehicle roll angle directly measured by the dual-antenna is also depicted (ground truth), $y_{meas}=\left[a_{ym},\phi_{exp},\dot{\phi }\right]$. Analyzing the results, we can observe that if the lateral acceleration and the roll rate are the only measurements fed to the DKF, $y_{meas}=\left[a_{ym},\dot{\phi }\right]$, the estimated vehicle roll angle is very noisy. Additionally, a quantitative analysis has been performed. The equation to calculate the norm error as a function of time is [27]:

(37) $$E_{t}=\frac{\varepsilon_{t}}{\sigma_{t}}$$

where,

(38) $$\begin{array}{c} \varepsilon_{t}^{2}=\int_{0}^{T}{\left(\phi_{\exp}-\phi_{est}\right)}^{2}dt\\ \sigma_{t}^{2}=\int_{0}^{T}{\left(\phi_{\exp}-\mu_{\exp}\right)}^{2}dt \end{array}$$

$\phi_{exp}$ represents the real vehicle roll angle obtained from the dual antenna, $\phi_{est}$ represents the vehicle roll angle obtained from estimator and $\mu_{exp}$ is the mean value of the vehicle roll angle obtained from the dual antenna during the period T. The norm errors for observers without and with “pseudo-roll angle” are 1.33 and 1.02, respectively, and the maximum errors for both observers are 0.138 rad and 0.096 rad, respectively. Therefore, results show that when the “pseudo-roll angle” obtained from the NN is an input to the DKF, $y_{meas}=\left[a_{ym},\phi_{NN},\dot{\phi }\right]$, the norm and maximum errors are reduced.

**Figure 6:**
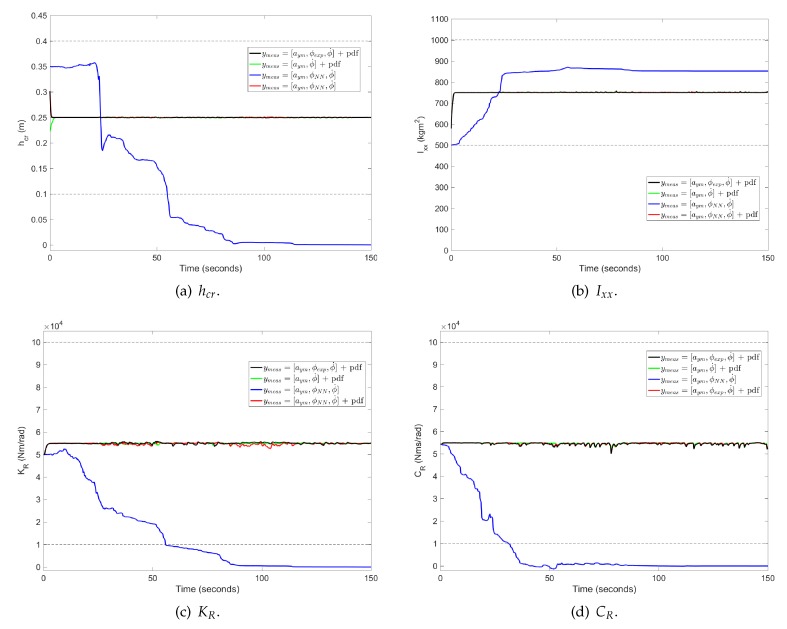
Parameter estimation for Case 1: (**a**) $h_{cr}$; (**b**) $I_{xx}$; (**c**) $K_{R}$ and (**d**) $C_{R}$. Black plot: $y_{meas}=\left[a_{ym},\phi_{exp},\dot{\phi }\right]$ + PDF. Green plot: $y_{meas}=\left[a_{ym},\dot{\phi }\right]$ + PDF. Blue plot: $y_{meas}=\left[a_{ym},\phi_{NN},\dot{\phi }\right]$. Red plot: $y_{meas}=\left[a_{ym},\phi_{NN},\dot{\phi }\right]$ + PDF.

An additional analysis is performed to prove the necessity for using a truncation DKF. The truncation is only performed for the parameter vector prediction, since results have shown a good estimation of the roll angle independently, of considering or not, a truncation in the state vector. This allows for simplification of the algorithm. In Figure 6, a comparison of the trend of vehicle’s parameters is shown. Represented in black, are the results obtained with a PDF-based truncation method when the input measurement is the roll angle obtained from the dual-antenna. These results are taken as our ground truth in order to validate the results obtained for the proposed algorithm. The blue plot represents the parameter’s trend when the PDF-based truncation method is not employed in the DKF. It is observed that the majority of estimated parameter values are outside the defined bounds, represented with dashed lines. Even $h_{cr}$ and $C_{R}$ take negative values which do not have physical meaning. Red and green plots represent the tendency of parameters when the PDF-based truncation method is used and the “pseudo-roll angle” is used an input or not, respectively. Both methods provide very similar results to the ground truth ones.

Even though the feeding of the “pseudo-roll angle”, obtained from the NN, to the DKF does not influence vehicle parameter’s estimation, this value is very useful for state estimation.

### 4.2. Case 2: DLC and J-Turn Manoeuvres

**Figure 7:**
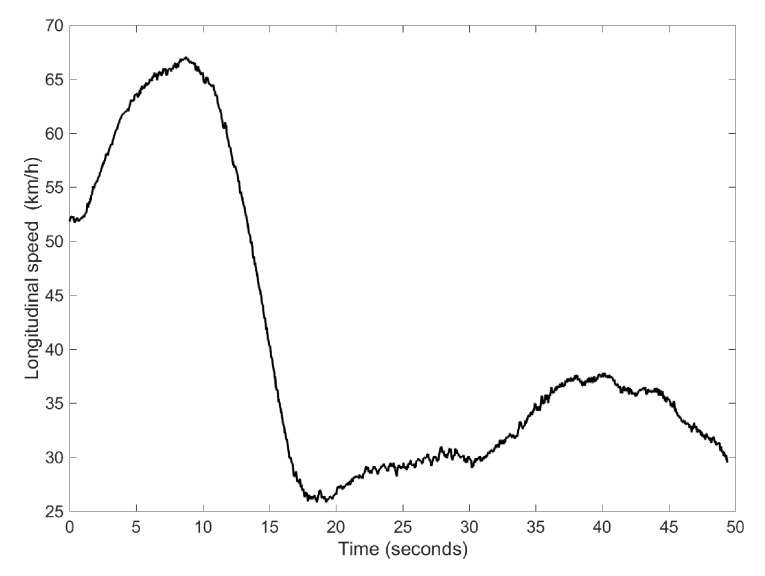
Speed profile for Case 2.

**Figure 8:**
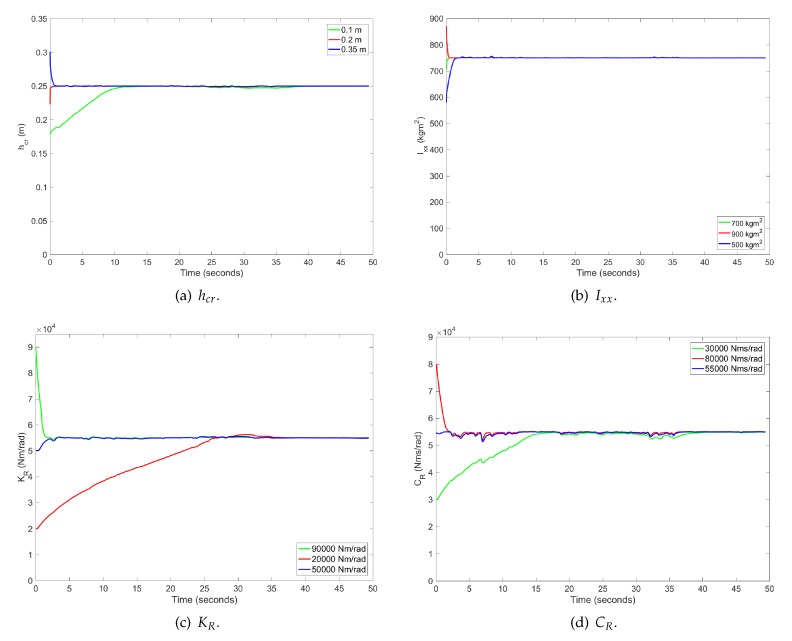
Parameter estimation for Case 2 for different initial values: (**a**) $h_{cr}$; (**b**) $I_{xx}$; (**c**) $K_{R}$ and (**d**) $C_{R}$.

The second test carried out is a double lane change (DLC) maneuver followed by a J-turn maneuver. The speed profile for this experiment is higher than in Case 1 and is shown in Figure 7. Figure 8 shows that the convergence of parameter values is independent of the considered initial values. The maximum time required for the parameters to reach stabilization is about 30 s. This information is important in order to know when the parameters have reached adequate values.

In this case, we want to prove the convergence of the proposed algorithm for different initial parameters values. In Table 1, the considered initial parameter values are given.

### 4.3. Case 3: Slalom and J-Turn Manoeuvres

**Figure 9:**
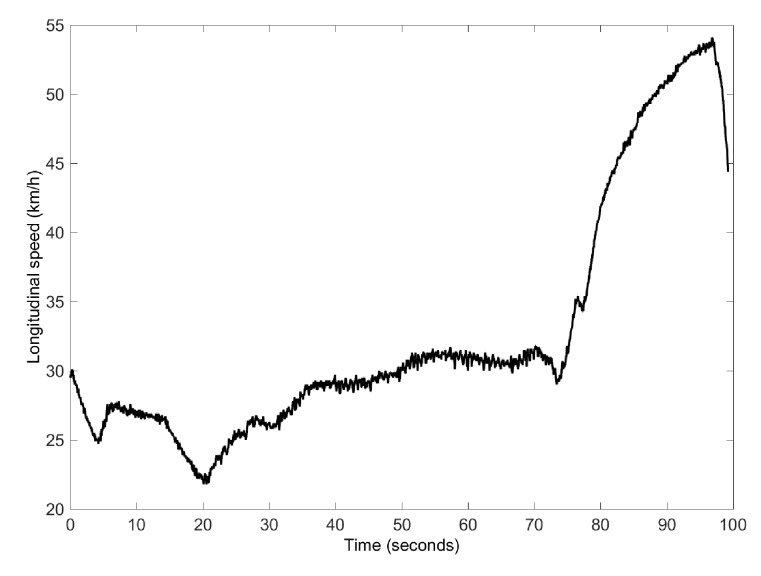
Speed profile for Case 3.

Finally, the third test is a combination of a slalom and a J-turn manoeuvre. This test case is employed to compare the proposed algorithm performance when using an estimated “pseudo-roll angle” derived from the NN with the estimated “pseudo-roll angle” obtained from suspension deflection measurements, as proposed by [6]. The vehicle speed profile is shown in Figure 9.

**Figure 10:**
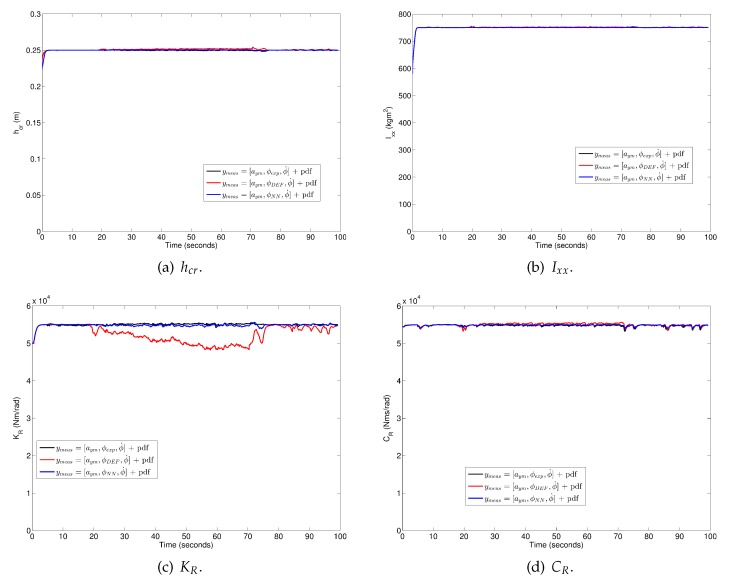
Parameter estimation for Case 3: (**a**) $h_{cr}$; (**b**) $I_{xx}$; (**c**) $K_{R}$ and (**d**) $C_{R}$. Black plot: $y_{meas}=\left[a_{ym},\phi_{exp},\dot{\phi }\right]$ + PDF. Blue plot: $y_{meas}=\left[a_{ym},\phi_{DEF},\dot{\phi }\right]$ + PDF. Red points: $y_{meas}=\left[a_{ym},\phi_{NN},\dot{\phi }\right]$ + PDF.

Figure 10 shows that parameters converge to the same values regardless of whether the “pseudo-roll angle” was obtained from the NN or from suspension deflection measurements, with the exception of parameter $K_{R}$, which is influenced by an inaccurate estimation of the vehicle roll angle, through the “pseudo-roll angle” derived from suspension deflection measurements.

**Figure 11:**
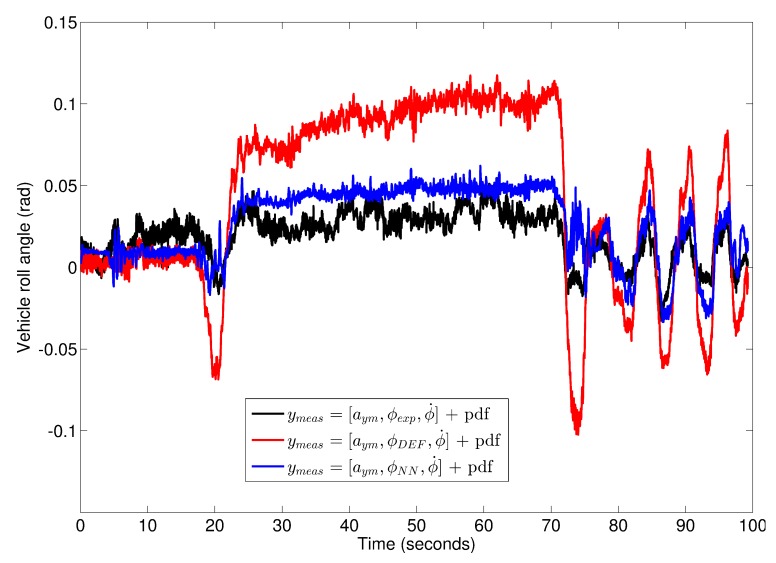
Vehicle roll angle for Case 3: Experimental data with dual antenna (black plot), estimated vehicle roll angle considering the “pseudo-roll angle” from suspension deflection (red points) and estimated vehicle roll angle considering the “pseudo-roll angle” from NN (blue plot).

Nevertheless, the vehicle roll angle is better estimated if the “pseudo-roll angle” is obtained from the NN and used as a measurement in the DKF, rather than employing the “pseudo-roll angle” derived from suspension deflection measurements as shown in Figure 11. The norm errors for observers with “pseudo-roll angle” from suspension deflection and NN are 3.28 and 1.05, respectively, and the maximum errors for both observers are 0.1 rad and 0.053 rad, respectively.

## 5. Conclusions

In this paper, an algorithm for the simultaneous on-line estimation of vehicle roll angle and vehicle parameters is proposed. This algorithm uses a PDF-based truncation method in combination with a DKF, to guarantee that both vehicle’s states and parameters are within bounds that have a physical meaning.

The proposed algorithm complies with the desired design criteria: it estimates, simultaneously and on-line, the vehicle’s states and parameters; it uses a simplified vehicle model in order to reduce complexity and computing time; it is useful in all kinds of environments (tunnels, urban and forested driving environments) due to the use of the “pseudo-roll angle” estimated from sensors installed on-board in current vehicles instead of GPS dual-antenna, and, finally, it takes into consideration both the measurements and model errors.

The proposed algorithm guarantees the convergence of vehicle parameter values regardless of the initial ones. Moreover, the PDF-based truncation method has only been applied in parameters vector, since experimental results have shown a good estimation of vehicle roll angle without the necessity of truncating the state vector.

The use of “pseudo-roll angle” obtained from the NN to be incorporated as a measurement in the DKF, has proved to be adequate. Finally, the advantage of the NN is that it takes into consideration the non-linearities of the system.

## Figures and Tables

**Table 1:** Initial parameters values for Case 2.

Parameter	Initial Values		
	Case 2.a	Case 2.b	Case 2.c
$h_{cr}$ (m)	0.1	0.2	0.35
$I_{xx}$ (kg m${}^{2}$)	700	900	500
$K_{R}$ (Nm/rad)	90,000	20,000	50,000
$C_{R}$ (Nms/rad)	30,000	80,000	55,000

## Abbreviations

The following abbreviations are used in this manuscript:

COG	Center of fravity
DLC	Double lane change
DKF	Dual Kalman filter
GPS	Global positioning system
IMU	Inertial measurement unit
NN	Neural networks
PDF	Probability density function

## Appendix A. Jordan Canonical Decomposition

Matrices $T_{i}$ and $W_{i}$ are obtained from the Jordan canonical decomposition. In this paper, both matrices are obtained using the matlab command:

(A1) $$\left[T_{i},W_{i}\right]=eig\left(P_{p,i}\left(k\right)\right)$$

## Appendix B. Gram–Schmidt Orthogonalization Algorithm

The Gram–Schmidt orthogonalization algorithm is given by the following procedure [16]:

1. For j=1, suppose that $S_{i}$ is a nxn matrix, where n is the number of estimate parameter, with rows $S_{i,j}$ (j=1,⋯,n):
  (A2) $$S_{i}={\left[S_{i,1},\cdots ,S_{i,n}\right]}^{T}$$
  The first row of $S_{i}$ is computed as:
  (A3) Si,1=DiT(k)·T·W1/2DiT(k)·Pp,i(k)·Di(k)12
2. For j=2,⋯,n:
  (a) Compute $S_{i,j}$:
    (A4) $$S_{i,j}=e_{j}-\sum_{m=1}^{k-1}\left(e_{m}^{T}\cdot S_{i,m}^{T}\right)\cdot S_{i,m}^{T}$$
    where $e_{j}$ is a vector that is an *n*-element column vector comprised entirely of zeros, except that its *k*th element is a 1.
  (b) if $S_{i,j}=0$, then replace it with:
    (A5) $$S_{i,j}=e_{1}-\sum_{m=1}^{k-1}\left(e_{1}^{T}\cdot S_{i,m}^{T}\right)\cdot S_{i,m}^{T}$$
  (c) Normalize $S_{i,j}$:
    (A6) $$S_{i,j}=\frac{S_{i,j}}{{∥S_{i,j}∥}_{2}}$$
